# Supplementary material for: Structure of the stress-related LHCSR1 complex determined by an integrated computational strategy
Source: Commun Biol. 2022 Feb 17;5:145. doi: 10.1038/s42003-022-03083-8 (PMC8854571; doi:10.1038/s42003-022-03083-8)
Supplement: Supplementary file 2 — Supplementary Information [file 42003_2022_3083_MOESM2_ESM.pdf]

Supplementary Information: Structure of the stress-related LHCSR1 complex determined by an integrated computational strategy

Ingrid Guarnetti Prandi[1, †], Vladislav Sláma[1, †], Cristina Pecorilla[1], Lorenzo Cupellini[1,*], and Benedetta Mennucci[1,*]

[1]Department of Chemistry and Industrial Chemistry, University of Pisa, 56124 Pisa, Italy
[*]Corresponding Author, e-mail: lorenzo.cupellini@unipi.it (LC), benedetta.mennucci@unipi.it (BM)

Supplementary Methods

Spectral densities The spectral density of the Chl excited states was modeled as a sum of overdamped Brownian oscillator and 48 high-frequency modes. The parameters for the spectral density were taken from the fitting of the fluorescence line narrowing experimental data for LHCII by Novoderezhkin et al.[1]. The spectral density of the Car S_2 excited state has been modeled by a sum of Brownian oscillator and two high frequency modes. The high frequency modes of the Cars were described by underdamped Brownian oscillator spectral density with the two most pronounced vibrational modes at $1050cm^{-1}$ and $1600cm^{-1}$, which corresponds to C-C and C=C stretch frequencies of the π-conjugated chain which are coupled to the optical transition[2]. The Huang-Rhys factors and corresponding reorganization energies λ of the individual vibrations and parameters of the Brownian oscillator were obtained from fitting the optical spectra of the isolated carotenoids in n-hexane as $\lambda_{1050} = 400cm^{-1}$ and $\lambda_{1600} = 1600cm^{-1}$. The low frequency modes were modeled with the overdamped Brownian oscillator spectral density with correlation time $\tau_c = 200fs$ and reorganization energy $\lambda = 250cm^{-1}$. Parameters of the low frequency part of the spectral density were obtained from fitting of the width of the experimental spectra. The comparison of the Car spectra from the fitted spectral density and the experimental one can be found in Supplementary Figure 22.

Site energies and static disorder For obtaining the pigment site energies we used a two-step approach, computing the excitation energies for the pigments in the protein environment and *in vacuo*, so as to separate the geometrical and environment contributions, as in our previous work[3]. The environment contribution is obtained as the difference between *in protein* and *in vacuo* excitation energies. The geometrical contribution arises from the distortion of bond lengths, angles, and dihedrals with respect to an optimized Chl structure. As previously found, a large variability in the excitation energy comes from the bond lengths[4]. As the bond length variability is connected with "fast" degrees of freedom, which are included in the spectral density, we adopted a statistical protocol to remove the bond-length contribution (ΔE^{bond}) from the excitation energies. The bond-length contribution was estimated through a multilinear fitting, as done in Ref. 4, and then averaged over all chlorophylls for all investigated frames. For each pigment in each frame, the bond-length contribution estimated from the fitting was subtracted from the excitation energy. The remaining geometrical contribution (distortion) was averaged only over the structures

of each pigment in each cluster because the average geometry could be statistically different in different pigments and conformations. The environmental contribution was computed for every single frame separately, which leads to correct thermal sampling of the excitation energy disorder. With this scheme, we (i) correct the intrinsic error of the MD simulation for the pigment bond lengths, (ii) exclude double-counting of bond length contribution to the energy disorder, and (iii) reduce the "noise" in the excitation energies coming only from the limited sampling of pigment geometries. The final corrected site energy for each pigment j in each configuration t reads:

$$E_{jt}^{\text{corrected}} = E_0 + \langle \Delta E^{\text{bond}} \rangle + \langle \Delta E_j^{\text{distortion}} \rangle + \Delta E_{jt}^{\text{env}} \tag{1}$$

where E_0 is the excitation energy of a reference pigment structure, $\langle \Delta E^{\text{bond}} \rangle$ is the average bond-length contribution, $\langle \Delta E_j^{\text{distortion}} \rangle$ is the pigment-specific average distortion contribution of pigment j, and $\Delta E_{jt}^{\text{env}}$ is the environment contribution for pigment j in configuration t. In this scheme, the Hamiltonian disorder is sampled directly from the distribution of ΔE^{env} rather than extracted from a predefined distribution.

The linear regression using all bond lengths explained more than 80% (computed as: $1 - \langle (E_{\text{bond}} - E)^2 \rangle / \langle (E - \bar{E})^2 \rangle$) of the variability of the Chls excitation energies *in vacuo* (Supplementary Figure 20). As in classical force fields the bond lengths should sample the same distribution, we expect the bond length contribution to average out. However, since this contribution has a major impact on the site energy fluctuations, it obscures the variability arising from the environment's conformation. Based on the linear regression, however, we were able to subtract the bond length contributions, and thus remove the large uncertainty in excitation energies coming from the fluctuations of bond lengths (Supplementary Figure 21). Site energies corrected according to Supplementary Equation 1, shown in Supplementary Figure 16, were used to build the disordered exciton Hamiltonian for each cluster.

We note that the site energies of the whole chlorophyll system were moved by -1381 cm^{-1} in order to account for the systematic error of the DFT approach and MD pigment geometries. The value of the site energy shift was independently determined in our previous study of the LHCII[3] and corrected to the smaller basis set used in this work to ensure the consistency between the studied light-harvesting antennas. The basis set correction was determined by comparison of the site energies of several selected chlorophylls computed for two replicas (100 frames) with M06-2X functional and 6-31+G(d) basis set, as used in our previous study of the LHCII[3], and the 6-31G(d) basis used in the current work. The site energies for the M06-2X/6-31+G(d) were systematically lower by 312 cm^{-1} with respect to the 6-31G(d) basis set. The site energy of carotenoids was moved by 1583 cm^{-1} for the same reason as chlorophylls (Supplementary Table 4). This value for the shift was determined by comparison of the experimental and QM energy gap between chlorophyll Q_y transition and carotenoid S_2 transition for isolated pigments in n-hexane. For CP29, Chl b transition energies were shifted by $+200$cm^{-1} to correct the intrinsic error of the TD-DFT method in estimation of the energy gap between Chl a and Chl b.

Supplementary Figures

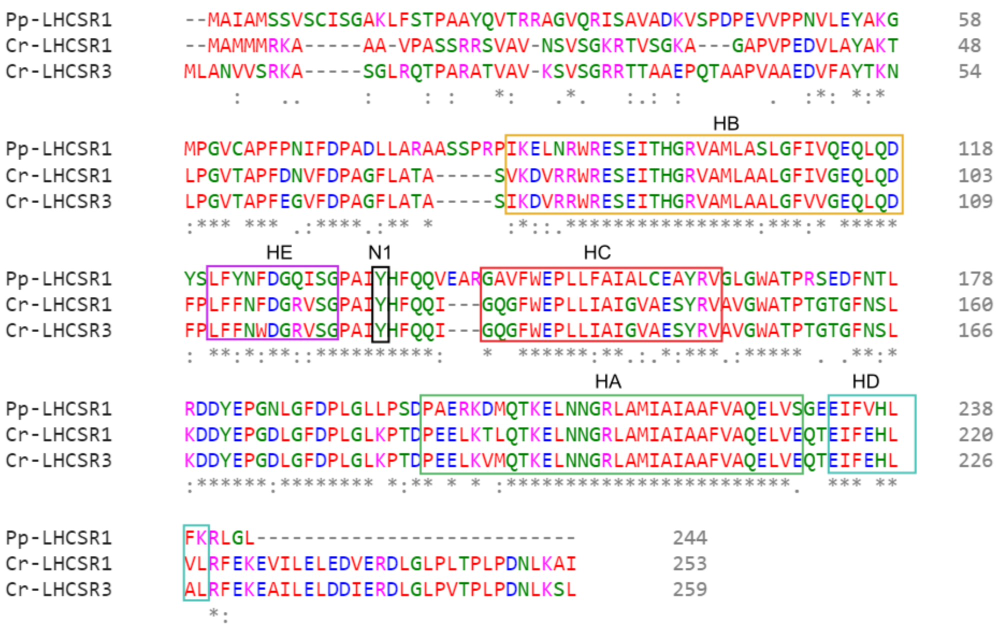

Supplementary Figure 1: Alignment of LHCSR sequences for LHCSR1 from *P. Patens* (Pp-LHCSR1) and LHCSR1 and LHCSR3 from *C. Reinhardtii* (Cr-LHCSR1, Cr-LHCSR3). Approximate positions of helices A-E are indicated by the square boxes. N1 indicates the conserved Tyr that constitutes the putative binding site for N1-Lut.

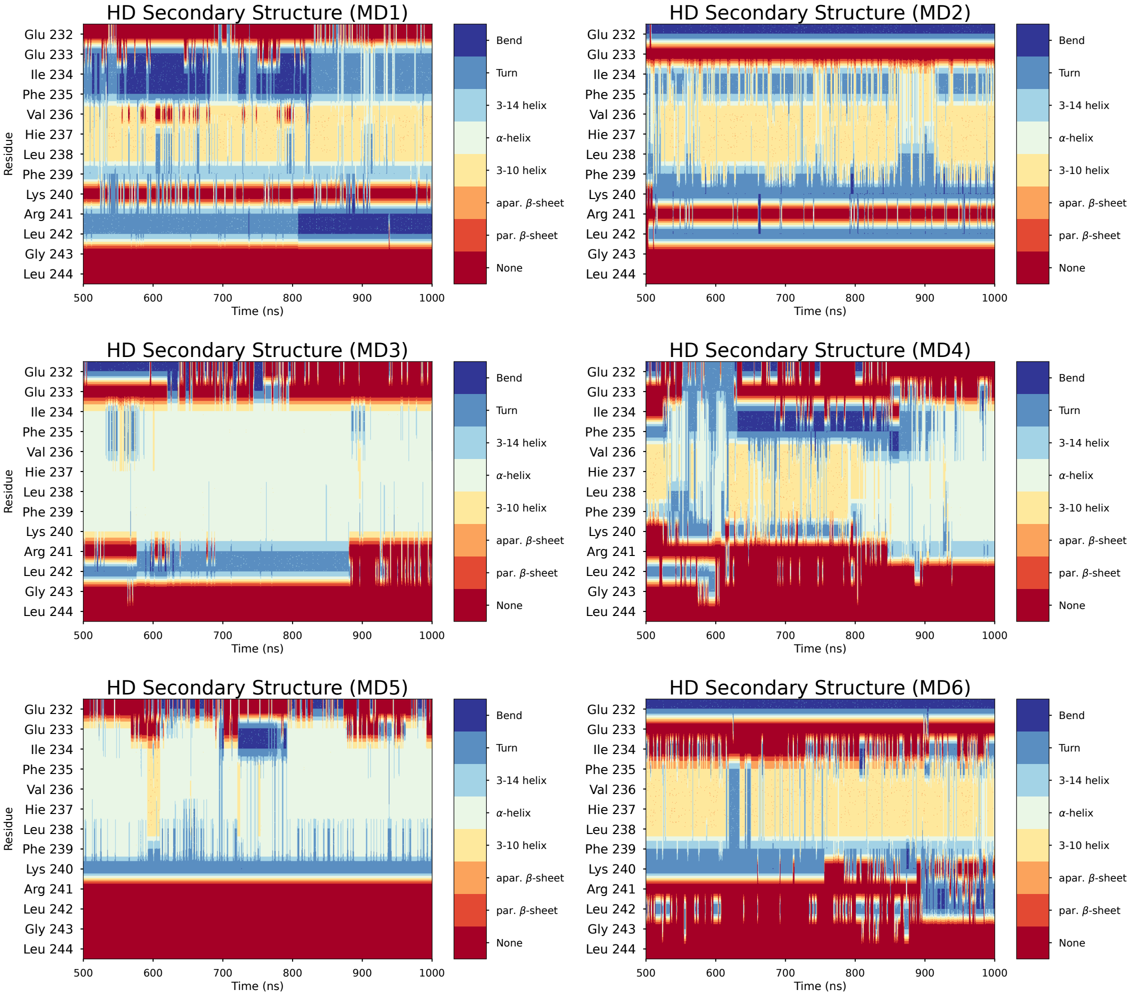

Supplementary Figure 2: Secondary structure in the Helix D region on refinement MDs 1-6. For the analysis we used one frame at ns from the second half of each trajectory, for a total of 500 structures for each replica.

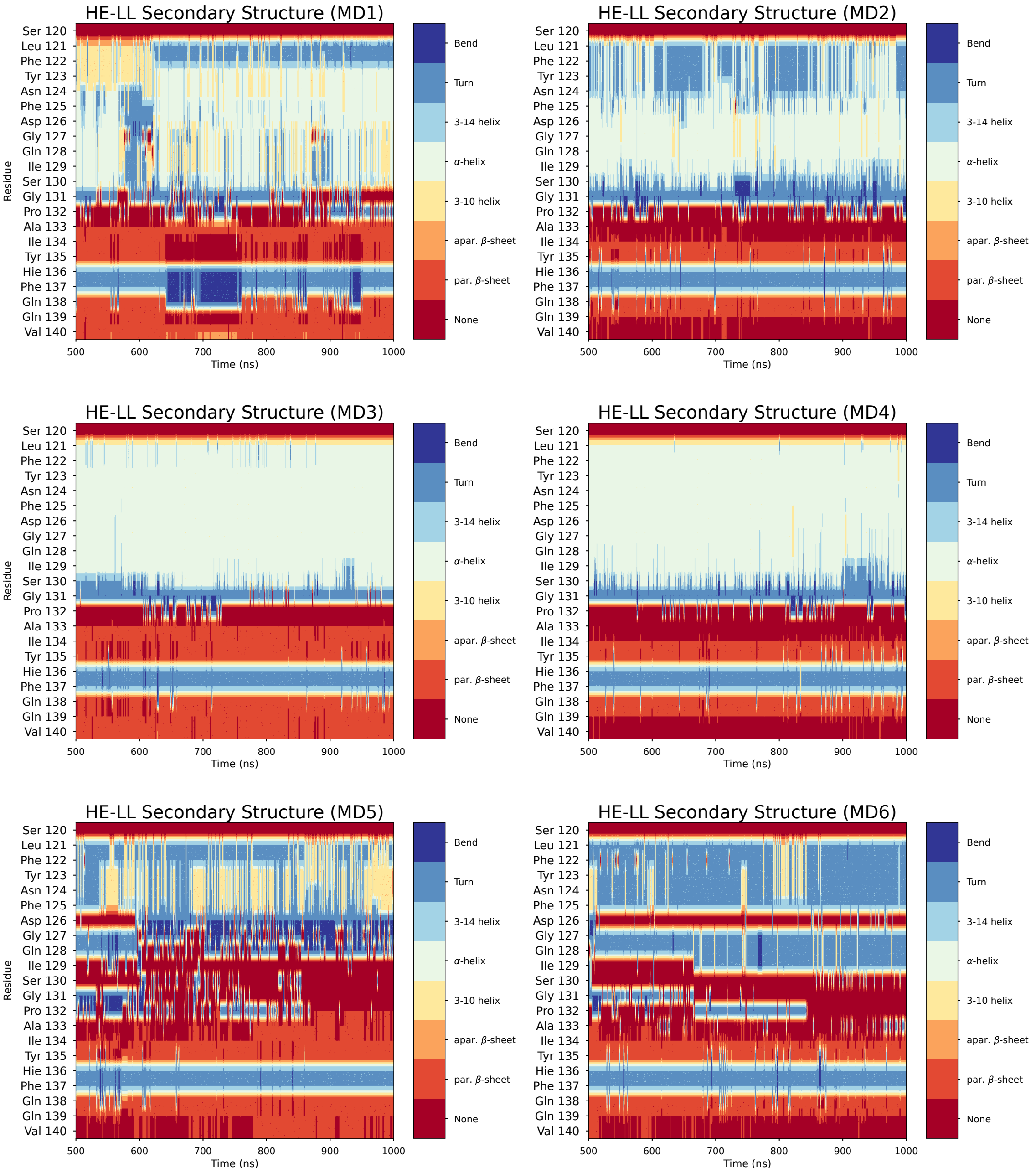

Supplementary Figure 3: Secondary structure in the Helix E For the analysis we used one frame at ns from the second half of each trajectory, for a total of 500 structures for each replica.

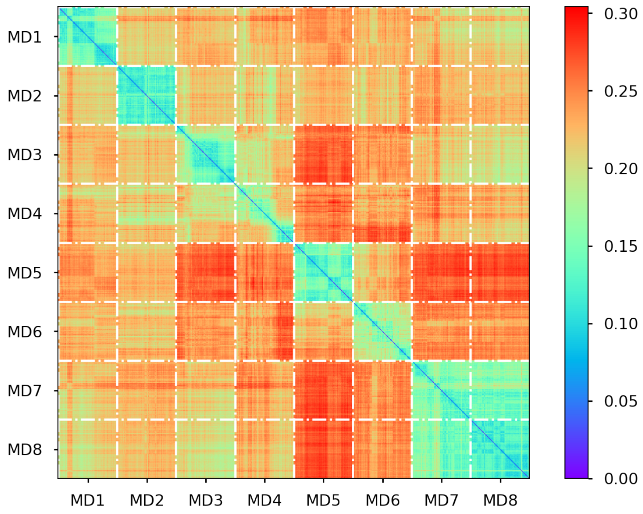

Supplementary Figure 4: RMSD in the Ramachandran torsional space between frames of the six replicas. 100 structures from the last 500 ns of each replica were included in this RMSD matrix. White lines separate the different replicas. Blue-green regions indicate pairs of structures that share a greater similarity.

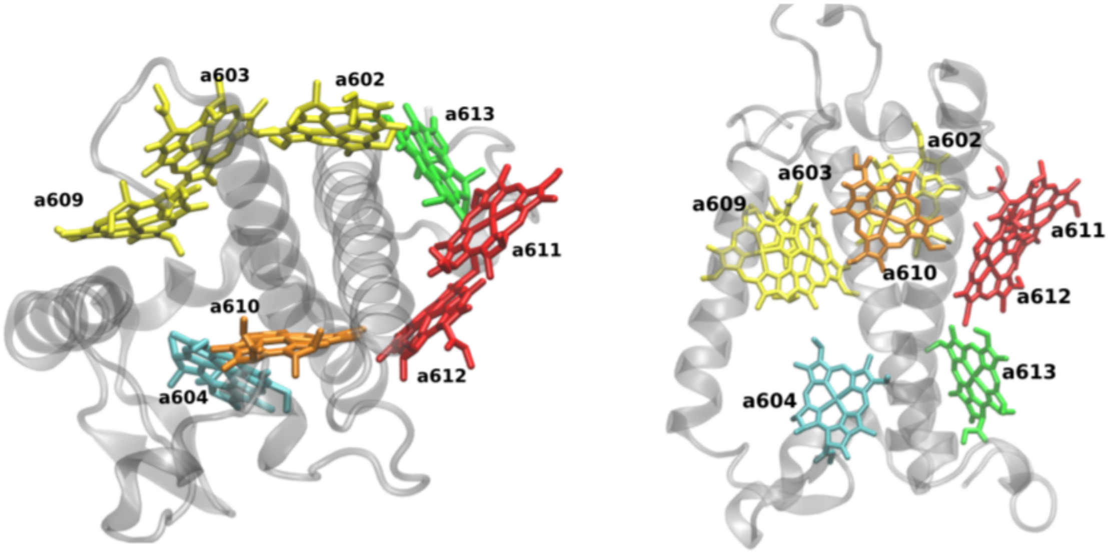

Supplementary Figure 5: Visualization of the LHCSR1 structure with the chlorophyll names

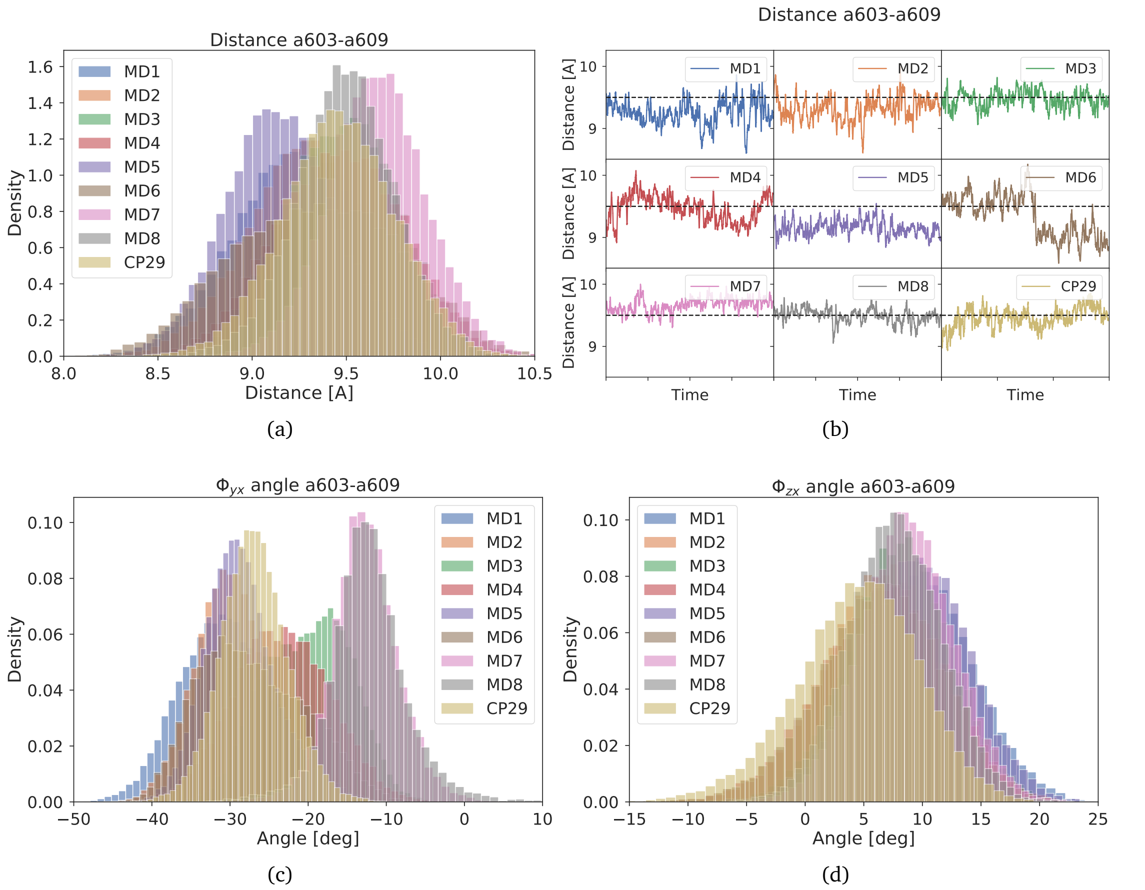

Supplementary Figure 6: a-b) Mutual distance between central Mg site of the chlorophyll a603 and a609 for the individual replicas (MD) of the LHCSR1 and CP29. For the time evolution, we plotted running average over 100 frames c) Mutual rotation of the chlorophylls in the plane of the chlorophyll conjugated ring. d) Mutual rotation of the chlorophylls in the plane perpendicular to the conjugated ring. For the analysis we used 20000 frames separated by 50 ps for each replica of the LHCSR1 MD simulation. For the CP29 30 000 frames separated by 50 ps were used for the analysis.

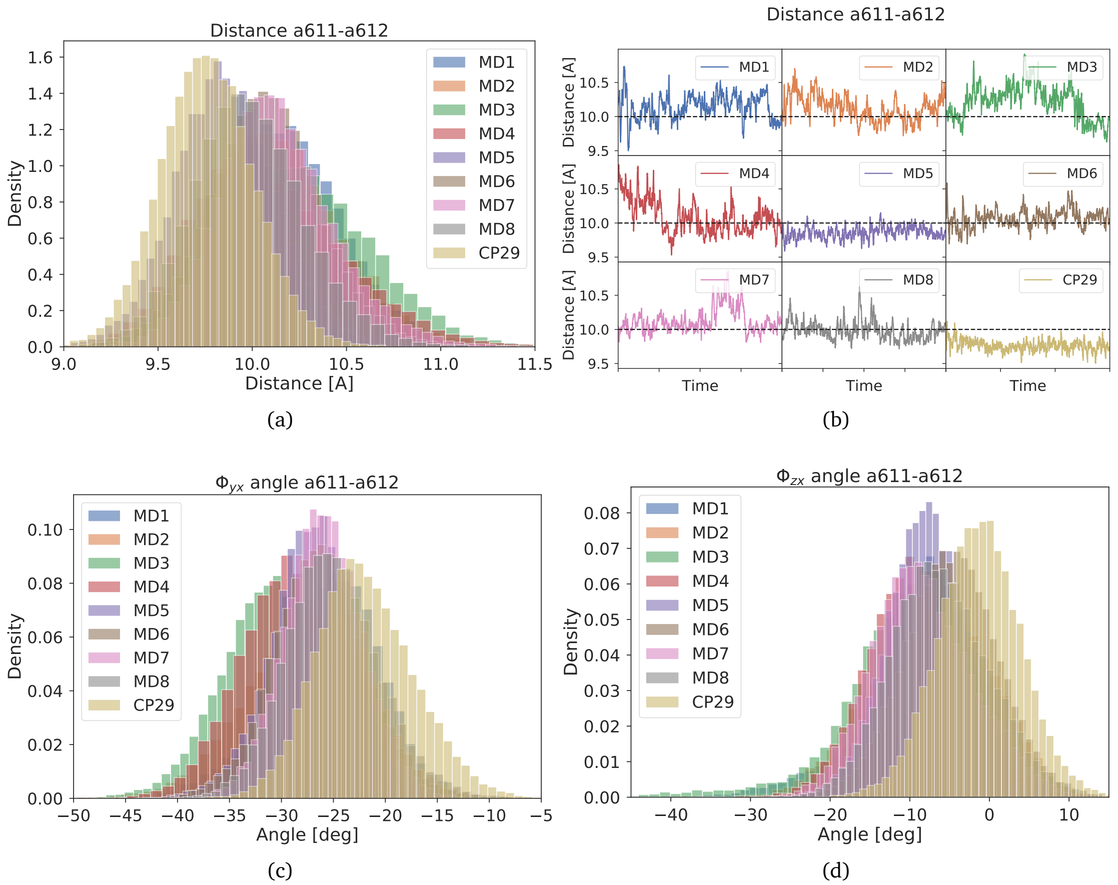

(a) (b) (c) (d)

Supplementary Figure 7: a-b) Mutual distance between central Mg site of the chlorophyll a611 and a612 for the individual replicas (MD) of the LHCSR1 and CP29. For the time evolution, we plotted running average over 100 frames c) Mutual rotation of the chlorophylls in the plane of the chlorophyll conjugated ring. d) Mutual rotation of the chlorophylls in the plane perpendicular to the conjugated ring. For the analysis we used 20000 frames separated by 50 ps for each replica of the LHCSR1 MD simulation. For the CP29 30 000 frames separated by 50 ps were used for the analysis.

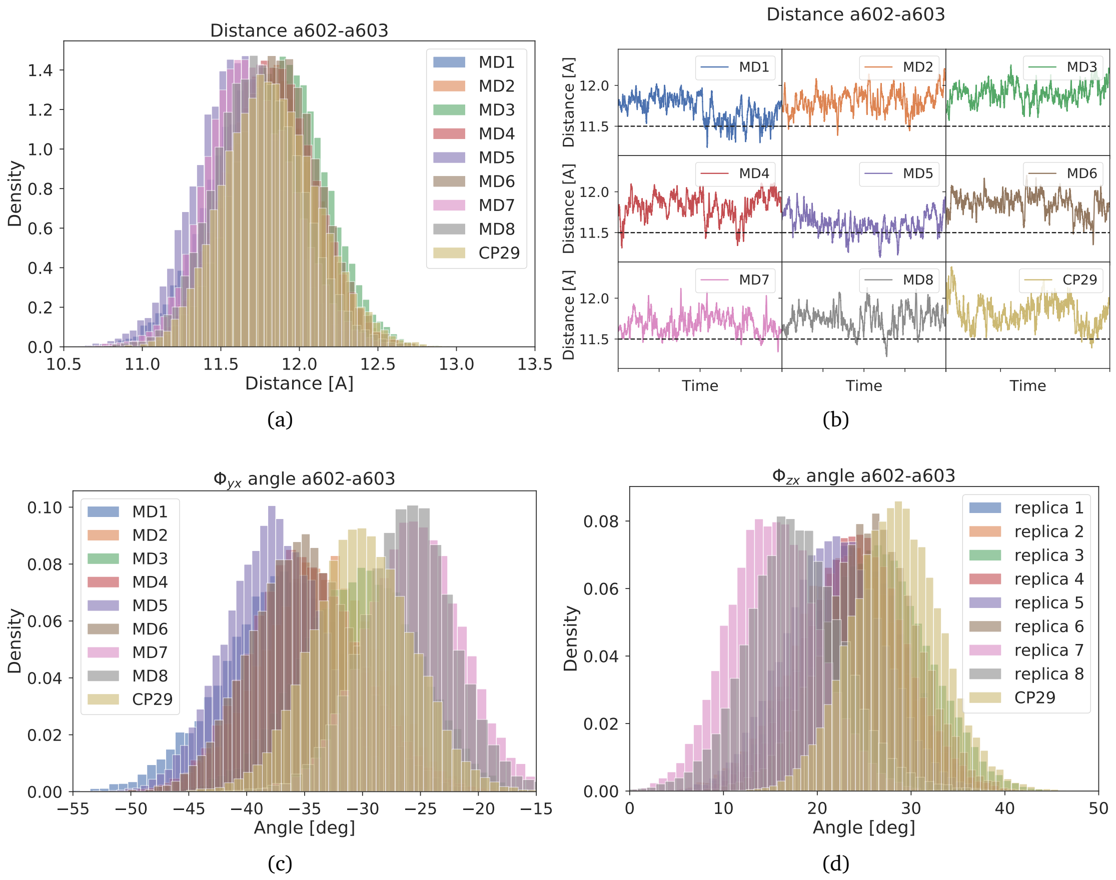

Supplementary Figure 8: a-b) Mutual distance between central Mg site of the chlorophyll a602 and a603 for the individual replicas (MD) of the LHCSR1 and CP29. For the time evolution, we plotted running average over 100 frames c) Mutual rotation of the chlorophylls in the plane of the chlorophyll conjugated ring. d) Mutual rotation of the chlorophylls in the plane perpendicular to the conjugated ring. For the analysis we used 20 000 frames separated by 50 ps for each replica of the LHCSR1 MD simulation. For CP29 300 00 frames separated by 50 ps were used for the analysis.

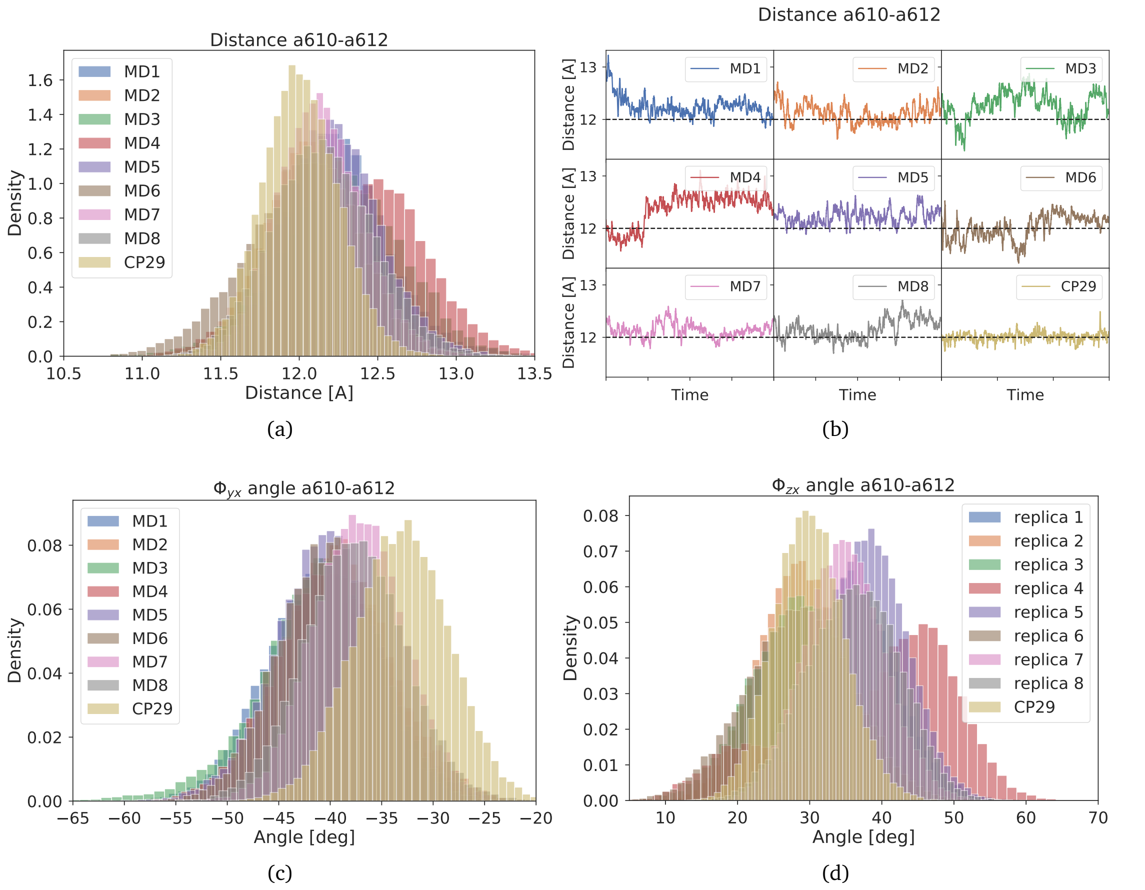

Supplementary Figure 9: a-b) Mutual distance between central Mg site of the chlorophyll a610 and a612 for the individual replicas (MD) of the LHCSR1 and CP29. For the time evolution we plotted running average over 100 frames. c) Mutual rotation of the chlorophylls in the plane of the chlorophyll conjugated ring. d) Mutual rotation of the chlorophylls in the plane perpendicular to the conjugated ring. For the analysis we used 20 000 frames separated by 5 0ps for each replica of the LHCSR1 MD simulation. For CP29 30 000 frames separated by 50ps were used for the analysis.

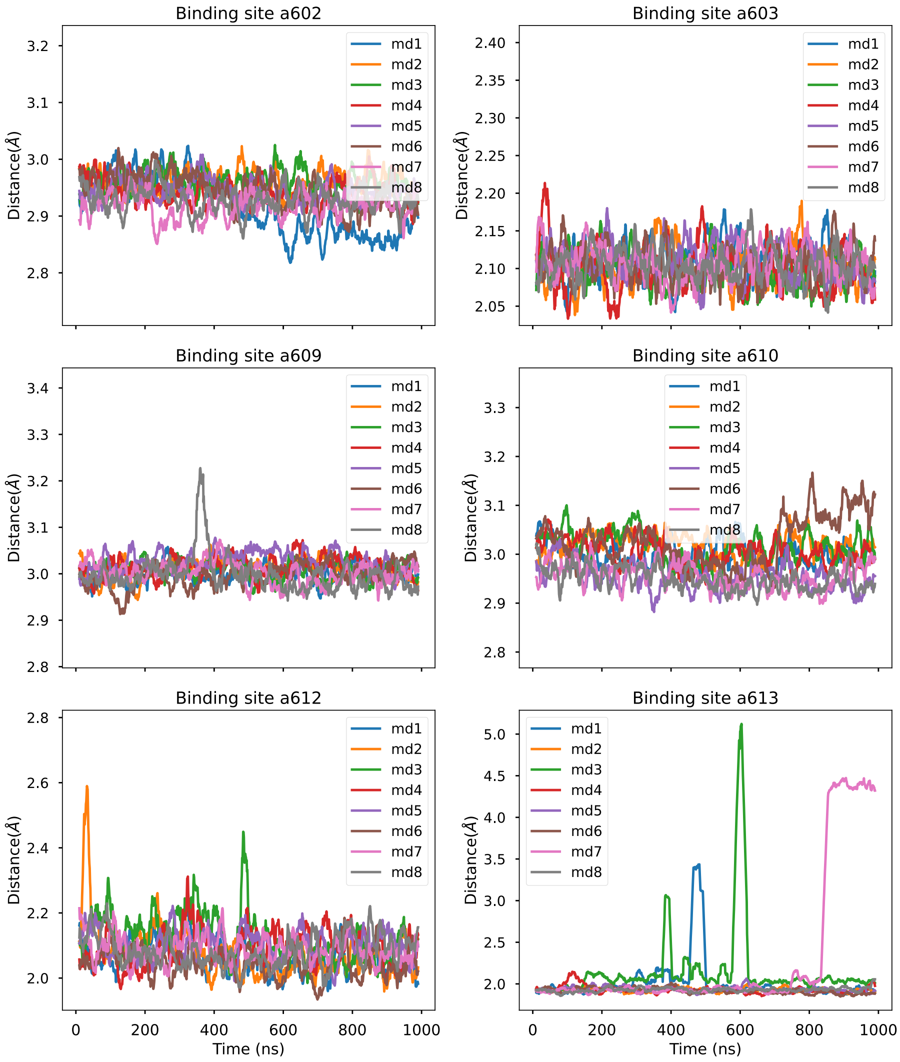

Supplementary Figure 10: Distances from the Mg to the binding site atom for the Chls coordinated by a protein residue, along the eight MD replicas.

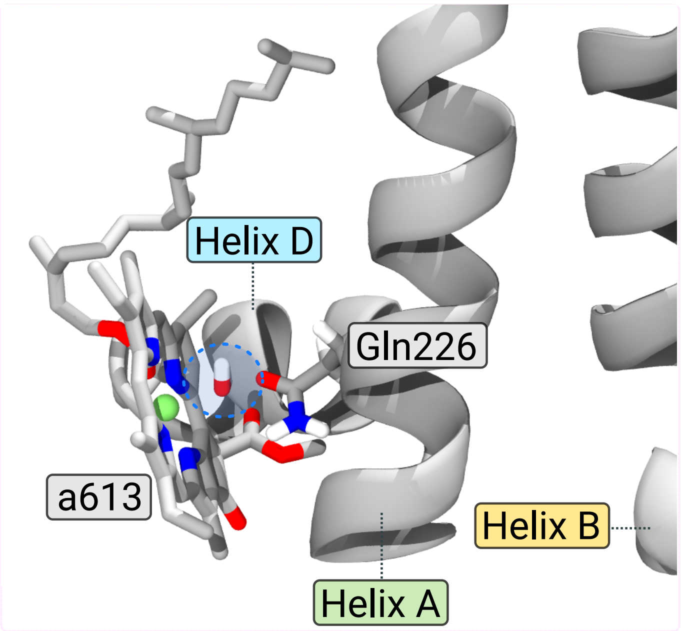

Supplementary Figure 11: Representative structure of Chl a613 in the last part of MD7, where the binding between the Mg atom and Gln226 occurs through a bridging water molecule.

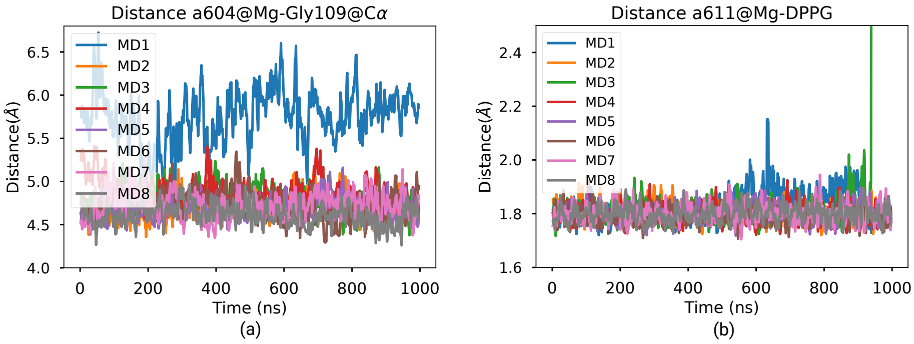

Supplementary Figure 12: (a) Distance from the Mg site of Chl a604 to the alpha carbon of the closest residue (Gly109) along the eight MD replicas. (b) Distance from the Mg site of Chl a611 to the closest oxygen of the DPPG lipid.

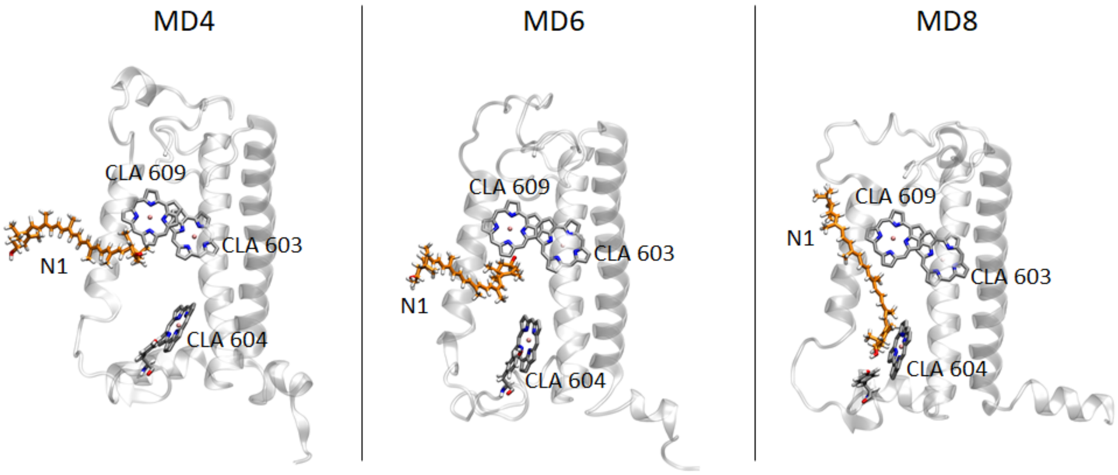

Supplementary Figure 13: Representative configurations showing the position of N1-Lut in MD4, MD6, and MD8. Chlorophylls a603 a064 and a609 are also shown, together with the binding Tyr.

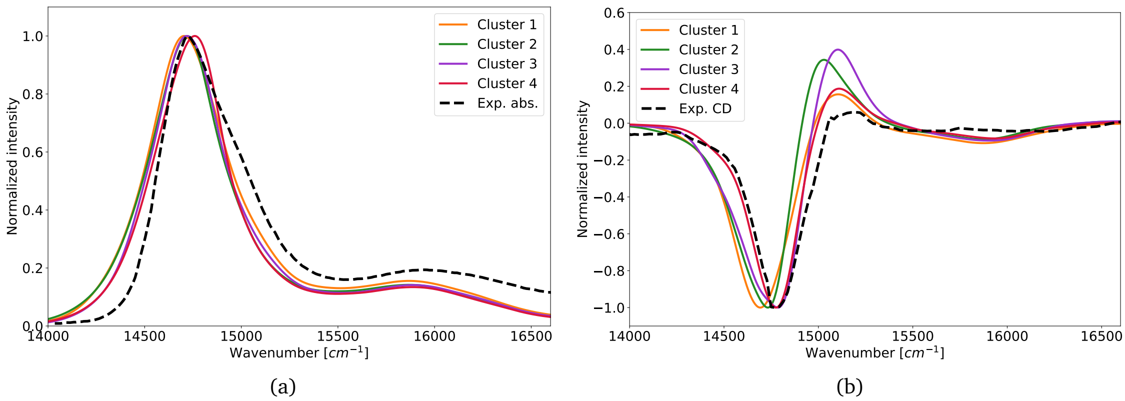

Supplementary Figure 14: a) Absorption and b) CD spectra at 283K for the individual clusters of the LHCSR1. Best agreement with the experimental spectra Ref 5 is obtained for the cluster 4 which is than used in the main text for all the comparison with the CP29. The CD spectra are normalized to the main negative peak of the cluster 4 of the LHCSR1 system. The spectra of cluster 1 were obtained by averaging over spectra of the 102 conformations (replicas MD1 and MD2), spectra of the cluster 2 over 102 conformations (replicas MD3 and MD4), spectra of cluster 3 over 51 conformations (replica MD5) and spectra of cluster 4 over 102 conformations (replicas MD7 and MD8).

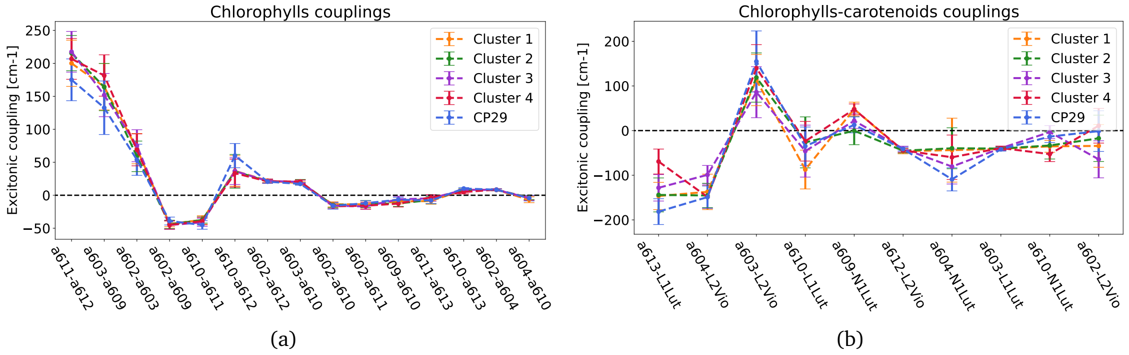

Supplementary Figure 15: Exciton couplings between individual pigments of the LHCSR1 compared with the couplings between corresponding pigments in the CP29. The couplings are ordered from the largest ones to the lowest (based on the cluster 1 of the LHCSR1) and the error bars correspond to the standard deviation of the coupling distribution. a) Exciton couplings between chlorophylls. b) Exciton couplings between chlorophylls and carotenoids. The mean values of the couplings and standard deviations of the coupling distribution were computed for the cluster 1 from 102 conformations (replicas MD1 and MD2), for the cluster 2 from 102 conformations (replicas MD3 and MD4), for the cluster 3 from 51 conformations (replica MD5) and for the cluster 4 from 102 conformations (replicas MD7 and MD8). The structures were selected equidistantly from the second half of the production run for each replica, one every 10 ns.

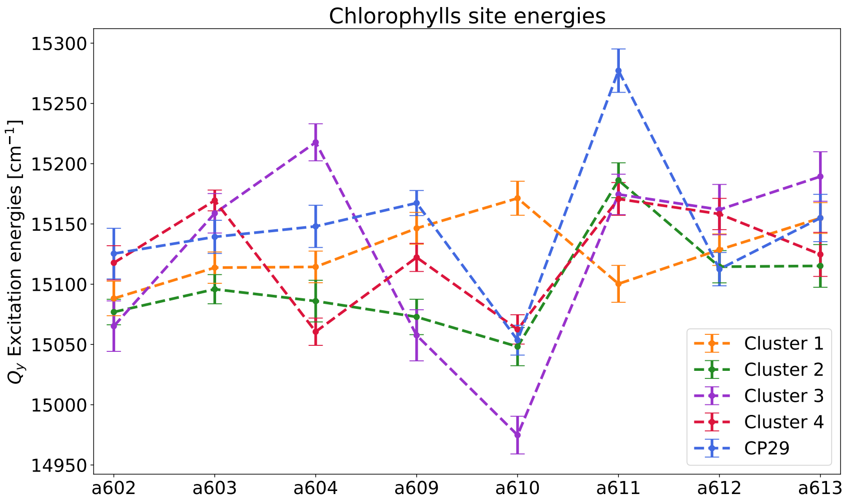

(a)

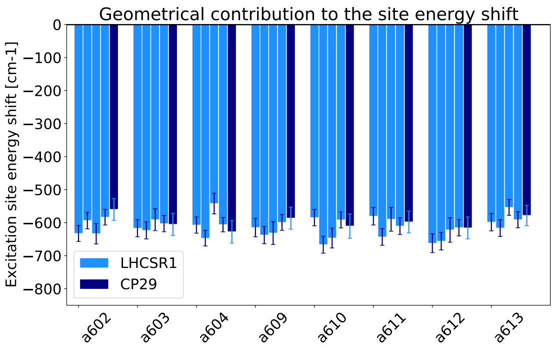

(b)

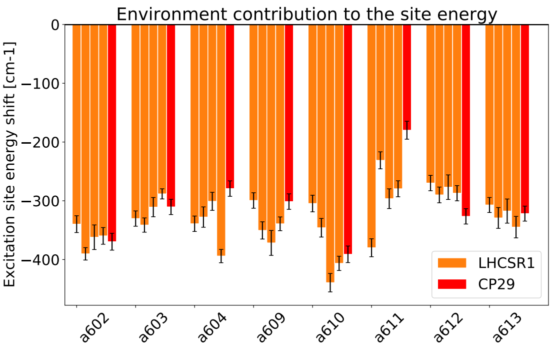

(c)

Supplementary Figure 16: a) TD-DFT M06-2X site energies for the individual clusters of the LHCSR1 compared with the ones for the CP29. The error bars correspond to the standard error of the mean value. The site energy is decomposed into the b) geometrical and c) environmental contribution. The geometrical contribution is defined as the difference of the vacuum excitation energy from the optimized pigment in vacuum. The environmental contribution is the difference between vacuum and MMPol excitation energies, where the protein and solvent environment are included. All the presented site energies are after removing the bond-length fluctuations as described in the Supplementary Methods. The mean values of the energies and their standard errors were computed for the cluster 1 from 102 conformations (replicas MD1 and MD2), for the cluster 2 from 102 conformations (replicas MD3 and MD4), for the cluster 3 from 51 conformations (replica MD5) and for the cluster 4 from 102 conformations (replicas MD7 and MD8). The structures were selected equidistantly from the second half of the production run for each replica, one every 10 ns.

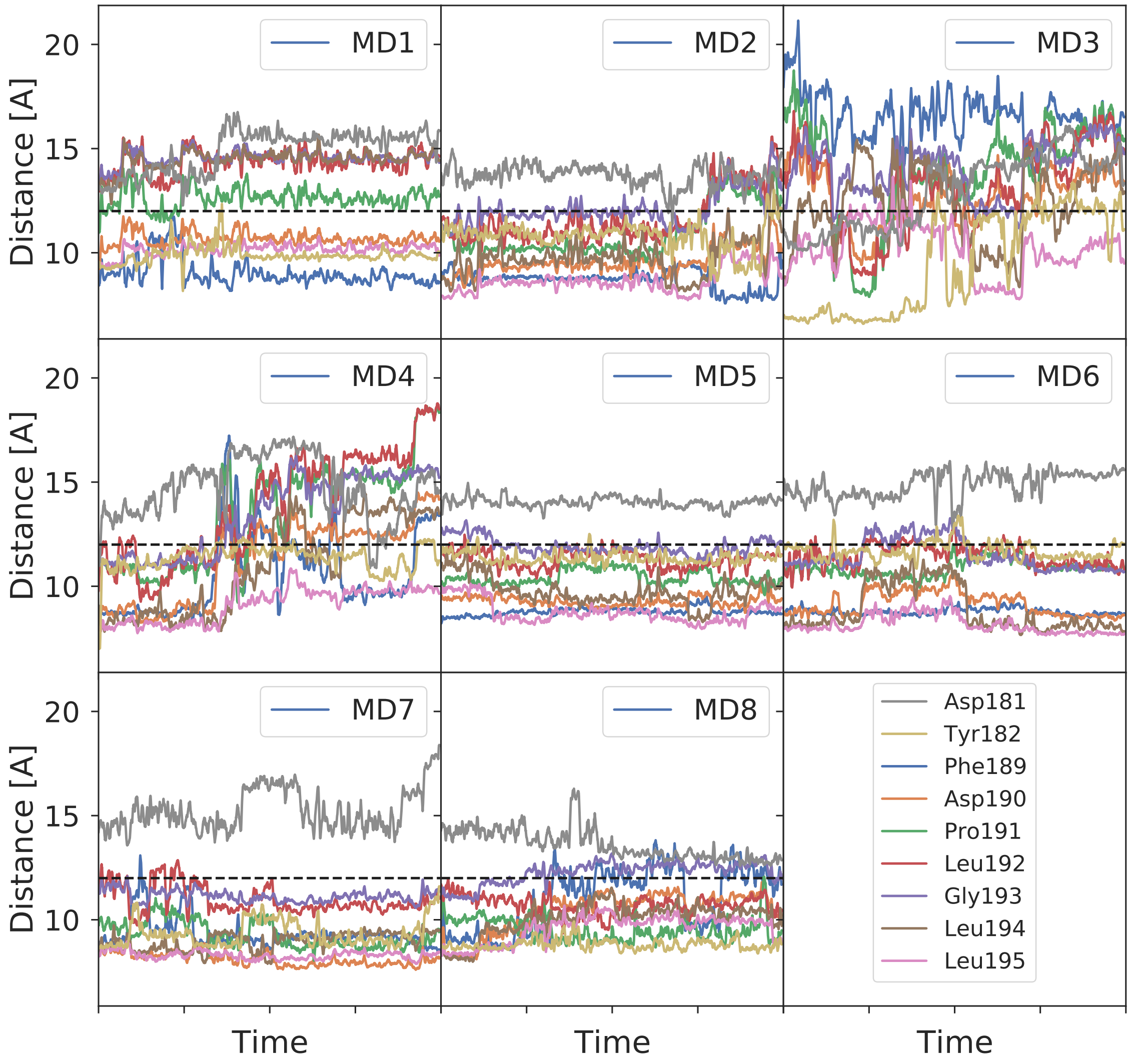

Supplementary Figure 17: Mutual distance between chlorophyll a610 and selected residues from the stromal loop for each replica of the LHCSR1.

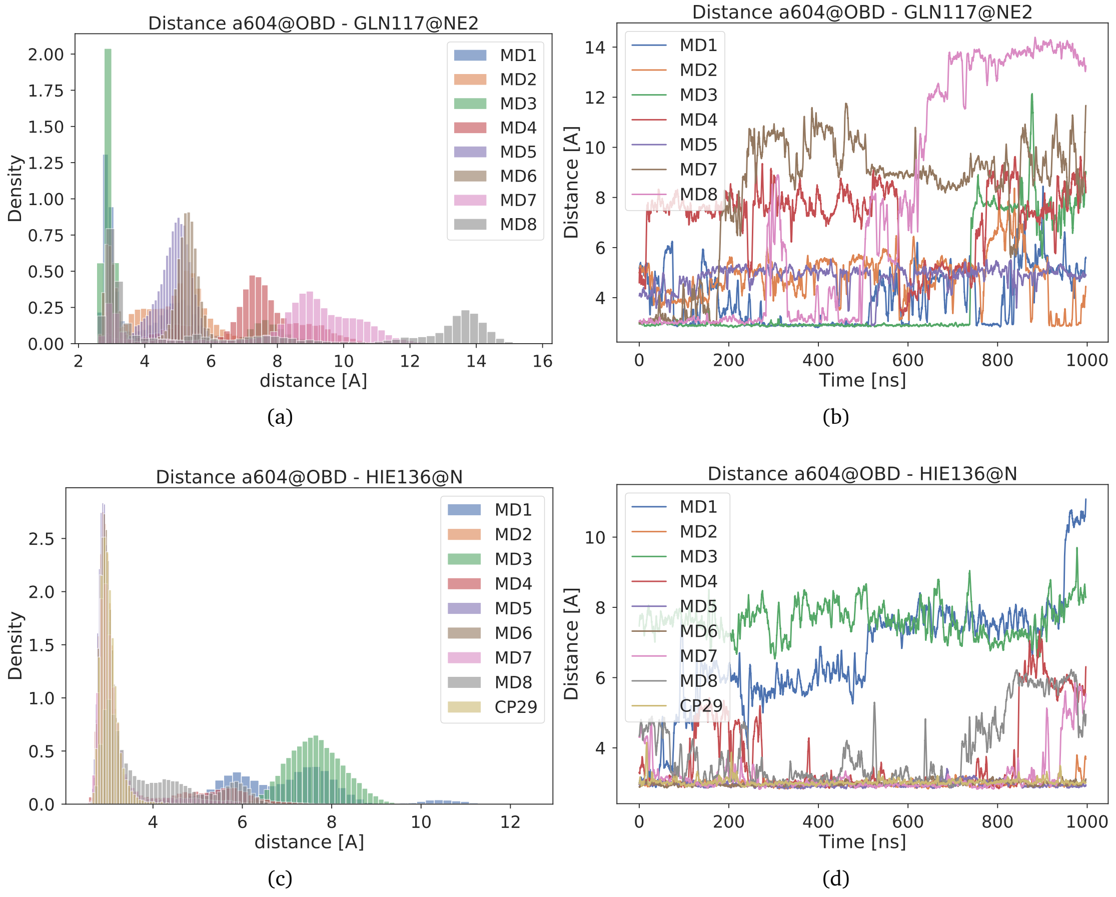

Supplementary Figure 18: Mutual distance between chlorophyll a604 OBD oxygen which is part of the π-conjugated system and hydrogen bonding residues a-b) Gln117 and c-d) His136 for the LHCSR1 and corresponding residue for the CP29. For the analysis we used 20 000 frames separated by 50 ps for each replica of the LHCSR1 MD simulation. For CP29 30 000 frames separated by 50 ps were used for the analysis.

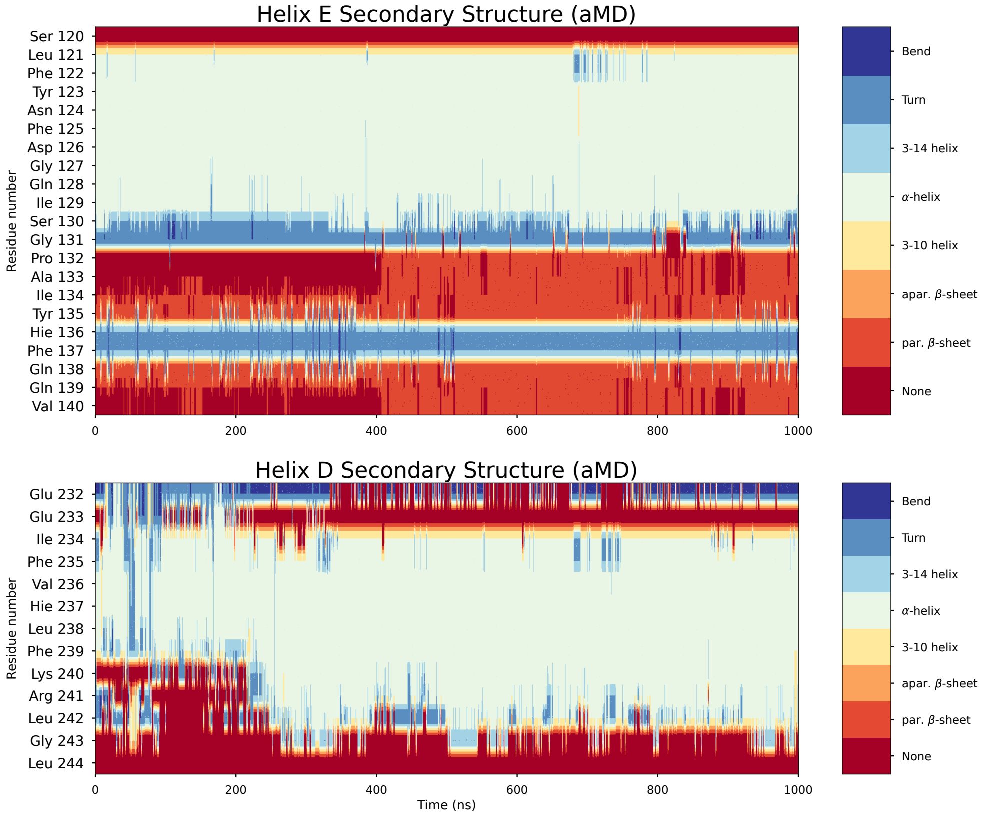

Supplementary Figure 19: Secondary structure of the lumenal side of the protein along aMD equilibration. For the analysis we used 1 frame for each ns, for a total of 1000 structures. We note here that after 300 ns, both helices E (residues 121 to 130) and D (residues 234 to 242) show an α-helix folding.

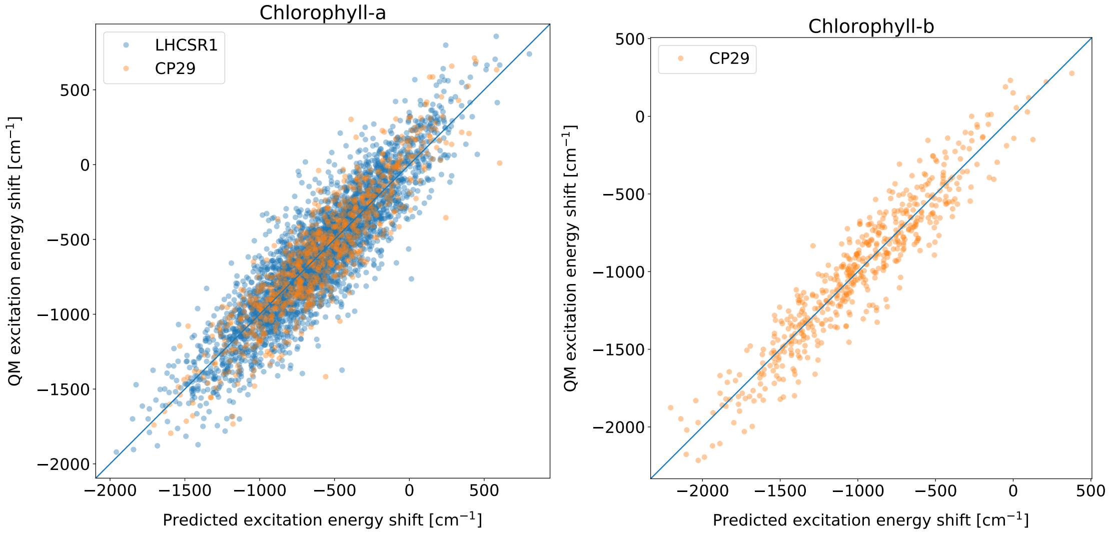

(a) (b)

Supplementary Figure 20: Comparison of the fitted (predicted) vacuum excitation energy with the full QM one. The excitation energy is given as a difference with respect to the optimized structure of the pigment. For the fitting only bond-lengths for the pi-conjugation chain were used. The fitted vacuum excitation energies correspond very well to the QM ones. The difference between the fitted and the full QM excitation energy is interpreted as an angular (distortion) contribution to the vacuum site energy. Fitting of the excitation energy for the chlorophyll-a was performed for 3264 conformations of the chlorophyll-a of the LHCSR. The another 790 conformations of the Chl-a from the CP29 was use as a test of the quality of the fit. The fit of the excitation energy for the chlorphyll-b was performed for 537 conformations of the chlorphyll-b of the CP29.

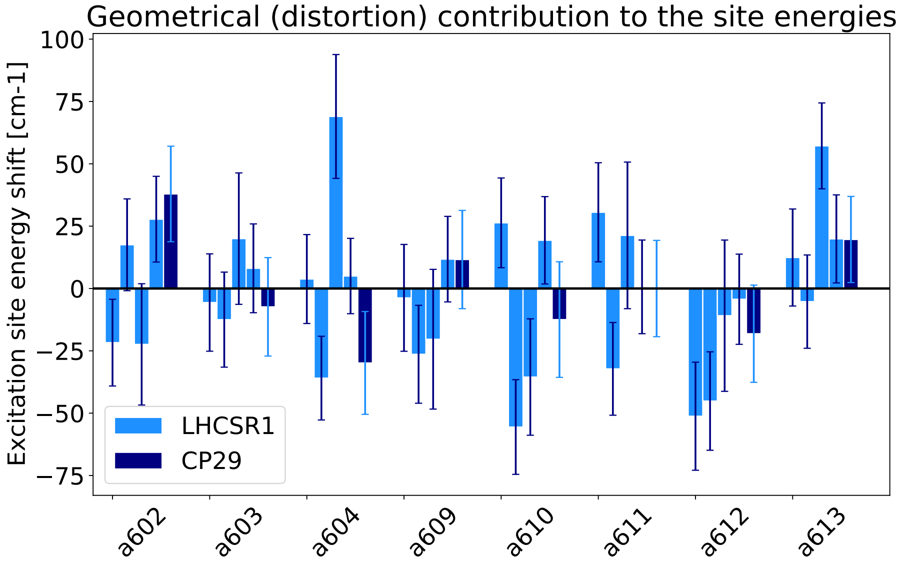

Supplementary Figure 21: The averaged distortion contribution to the excitation energy for the LHCSR1 clusters and the CP29. The distortion contribution was computed as the difference of the predicted excitation energy and the full QM vacuum excitation energy, which was then averaged over the individual clusters. The error bars correspond to the standard error of the mean value. The mean values of the energies and their standard errors were computed for the cluster 1 from 102 conformations (replicas MD1 and MD2), for the cluster 2 from 102 conformations (replicas MD3 and MD4), for the cluster 3 from 51 conformations (replica MD5) and for the cluster 4 from 102 conformations (replicas MD7 and MD8). The structures were selected equidistantly from the second half of the production run for each replica, one every 10 ns.

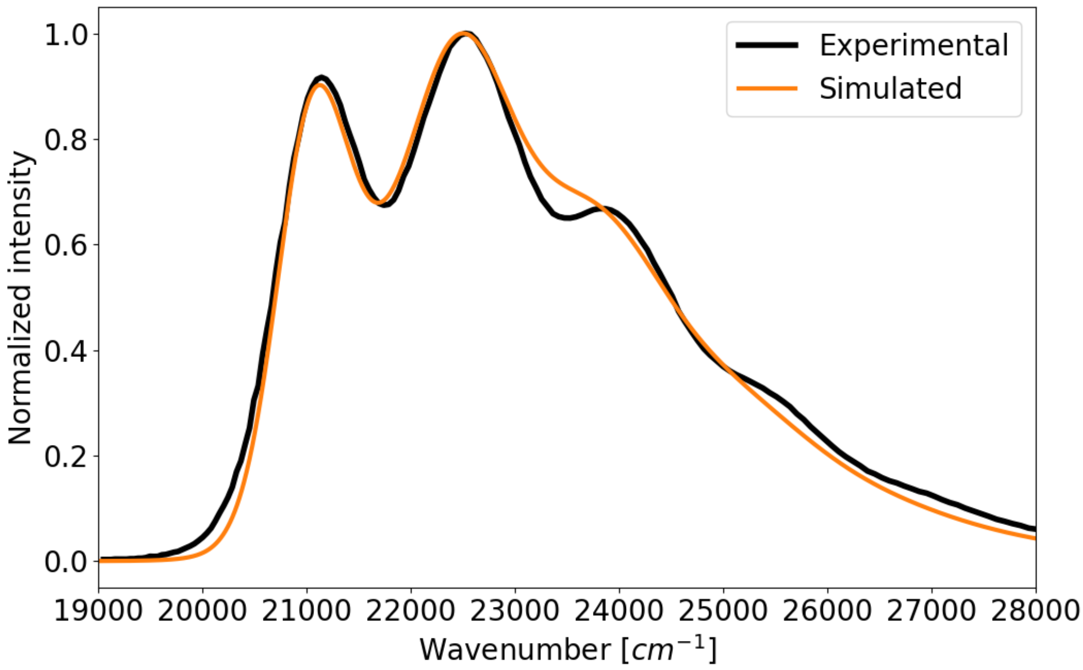

Supplementary Figure 22: Comparison of the simulated and experimental[6] spectra of the Lutein in n-hexane which have the optical permitivity close to the one in the protein.

Supplementary Tables

	a602	a603	a604	a609	a610	a611	a612	a613	L1-Lut	L2-Vio	N1-Lut
a602	15118	69	9	-45	-17	-16	21	-4	16	11	-2
a603	69	15170	-3	182	21	1	-3	6	-39	140	-17
a604	9	-3	15061	-6	-4	-4	4	3	14	-149	-60
a609	-45	182	-6	15122	-13	5	0	-3	17	-3	48
a610	-17	21	-4	-13	15062	-39	33	5	-24	14	-53
a611	-16	1	-4	5	-39	15171	207	-3	13	13	9
a612	21	-3	4	0	33	207	15158	0	21	-44	-33
a613	-4	6	3	-3	5	-3	0	15125	-69	18	-5
L1-Lut	16	-39	14	17	-24	13	21	-69	20202	-62	-27
L2-Vio	11	140	-149	-3	14	13	-44	18	-62	20180	106
N1-Lut	-2	-17	-60	48	-53	9	-33	-5	-27	106	19621

Supplementary Table 1: Exciton Hamiltonian of the LHCSR1 calculated within QM/MMpol approach at M062x/6-31G(d) level. All the values have been obtained as averages on the configurations extracted from MD and cluster 4, which represents the most equilibrated structure. The site excitation energies were shifted by -1381 cm^{-1} and carotenoids by +1583 cm^{-1}. All values are in cm^{-1}

	a601	a602	a603	a604	b606	b607	b608	a609	a610	a611	a612	a613	L1-Lut	L2-Vio	N1-Nex
a601	15146	13	-5	0	0	0	0	0	1	30	-6	1	3	-36	0
a602	13	15125	54	8	7	8	-8	-39	-16	-12	21	-4	9	-1	-10
a603	-5	54	15139	-4	-8	4	3	133	17	0	-3	1	-42	155	0
a604	0	8	-4	15148	123	32	-4	-6	-4	-3	1	1	8	-149	-109
b606	0	7	-8	123	15402	35	-4	1	-3	0	2	1	0	-83	-104
b607	0	8	4	32	35	15445	-5	-13	0	0	2	3	-5	-98	-15
b608	0	-8	3	-4	-4	-5	15420	46	79	7	-1	0	-8	-9	36
a609	0	-39	133	-6	1	-13	46	15167	-7	4	-1	-4	11	14	13
a610	1	-16	17	-4	-3	0	79	-7	15054	-45	60	10	-35	15	-13
a611	30	-12	0	-3	0	0	7	4	-45	15277	175	-8	4	16	8
a612	-6	21	-3	1	2	2	-1	-1	60	175	15112	1	54	-41	-18
a613	1	-4	1	1	1	3	0	-4	10	-8	1	15155	-181	4	-4
L1-Lut	3	9	-42	8	0	-5	-8	11	-35	4	54	-181	20167	-74	-12
L2-Vio	-36	-1	155	-149	-83	-98	-9	14	15	16	-41	4	-74	20323	101
N1-Nex	0	-10	0	-109	-104	-15	36	13	-13	8	-18	-4	-12	101	20737

Supplementary Table 2: Exciton Hamiltonian of the CP29 calculated within QM/MMpol approach at M062x/6-31G(d) level. All the values have been obtained as averages on the configurations extracted from MD in Ref 7. The site excitation energies of the chlorophylls were shifted by -1381 cm^{-1} and carotenoids by +1583 cm^{-1}. All values are in cm^{-1}.

exciton n.	LHCSR1	CP29
1	14910	14929
2	14947	14992
3	15054	15088
4	15085	15095
5	15125	15146
6	15153	15151
7	15329	15161
8	15371	15277
9		15390
10		15404
11		15445
12		15489
13	19603	20143
14	20139	20340
15	20274	20770

Supplementary Table 3: TD-DFT M06-2X/MMPol exciton energies (in cm^{-1}) determined from MD structures for the LHCSR1 cluster 4 and CP29. The exciton energies were computed from the averaged Hamiltonian and therefore represent only approximate exciton energies to the ones used for the optical spectra calculation, which were computed separately for the Hamiltonian of each cluster and frame.

	exp. peak position [cm^{-1}]	m06-2X [cm^{-1}]
Chl-a	15105	17085
Lutein	21120	20261
Violaxanthin	21277	20559

Supplementary Table 4: Comparison of the QM and experimental excitation energies for the LHCSR1 pigments in n-hexane. The TD-DFT energy gap between Chl-a and Cars computed with M06-2X functional is underestimated by ~3000 cm^{-1}. Therefore the Cars are for the absorption spectra calculation all consistently shifted by ~3000 cm^{-1} to get the correct experimental energy gap between Chl-a and Cars. This is needed to correctly evaluate the effect of the carotenoids on the optical spectra of both LHCSR1 and CP29.

Supplementary References

[1] Novoderezhkin, V. I., Palacios, M. A., van Amerongen, H. & van Grondelle, R. Energy-transfer dynamics in the LHCII complex of higher plants: modified redfield approach. *J. Phys. Chem. B* **108**, 10363–10375 (2004).

[2] Polívka, T. & Sundström, V. Ultrafast dynamics of carotenoid excited states - from solution to natural and artificial systems. *Chemical Reviews* **104**, 2021–2072 (2004). PMID: 15080720.

[3] Sláma, V., Cupellini, L. & Mennucci, B. Exciton properties and optical spectra of light harvesting complex II from a fully atomistic description. *Phys. Chem. Chem. Phys.* **22**, 16783–16795 (2020).

[4] Cardoso Ramos, F., Nottoli, M., Cupellini, L. & Mennucci, B. The molecular mechanisms of light adaption in light-harvesting complexes of purple bacteria revealed by a multiscale modeling. *Chem. Sci.* **2**, 9650–9662 (2019).

[5] Pinnola, A. *et al.* Functional modulation of LHCSR1 protein from physcomitrella patens by zeaxanthin binding and low pH. *Scientific Reports* **7**, 11158 (2017).

[6] Jeffrey, S., Mantoura, R. & Wright, S. *Phytoplankton pigments in oceanography: guidelines to modern methods.* (Cambridge University Press, Paris, 1997).

[7] Lapillo, M., Cignoni, E., Cupellini, L. & Mennucci, B. The energy transfer model of nonphotochemical quenching: Lessons from the minor cp29 antenna complex of plants. *Biochimica et Biophysica Acta (BBA) - Bioenergetics* **1861**, 148282 (2020).
