# Supplementary material for: Structure of the stress-related LHCSR1 complex determined by an integrated computational strategy
Source: Commun Biol. 2022 Feb 17;5:145. doi: 10.1038/s42003-022-03083-8 (PMC8854571; doi:10.1038/s42003-022-03083-8)
Supplement: Supplementary file 3 — Description of Additional Supplementary Files [file 42003_2022_3083_MOESM3_ESM.pdf]

Description of Additional Supplementary Files

File name: Supplementary Data 1
Description: Representative structures for clusters 1-4

File name: Supplementary Data 2
Description: Source Data for Figure 3

File name: Supplementary Data 3
Description: Source Data for Figure 4

File name: Supplementary Data 4
Description: Source Data for Figure 5 and Supplementary Tables 1-3

File name: Supplementary Data 5
Description: Source Data for Figure 6

File name: Supplementary Data 6
Description: Source Data for Figure 7

File name: Supplementary Data 7
Description: Source Data for Supplementary Figures 2 and 3

File name: Supplementary Data 8
Description: Source Data for Supplementary Figure 4

File name: Supplementary Data 9
Description: Source Data for Supplementary Figures 6-9

File name: Supplementary Data 10
Description: Source Data for Supplementary Figures 10 and 12

File name: Supplementary Data 11
Description: Source Data for Supplementary Figure 14

File name: Supplementary Data 12
Description: Source Data for Supplementary Figures 15 and 16

File name: Supplementary Data 13
Description: Source Data for Supplementary Figures 17 and 18

File name: Supplementary Data 14
Description: Source Data for Supplementary Figure 19

File name: Supplementary Data 15
Description: Source Data for Supplementary Figure 20 and 21

File name: Supplementary Data 16
Description: Source Data for Supplementary Figure 22

File name: Supplementary Data 16
Description: Source Data for Supplementary Table 4
