# Supplementary material for: Structure of the stress-related LHCSR1 complex determined by an integrated computational strategy
Source: Commun Biol. 2022 Feb 17;5:145. doi: 10.1038/s42003-022-03083-8 (PMC8854571; doi:10.1038/s42003-022-03083-8)
Supplement: Supplementary file 21 — Reporting Summary [file 42003_2022_3083_MOESM21_ESM.pdf]

nature research

Corresponding author(s): Lorenzo Cupellini
Benedetta Mennucci

Last updated by author(s): Jan 5, 2022

Reporting Summary

Nature Research wishes to improve the reproducibility of the work that we publish. This form provides structure for consistency and transparency in reporting. For further information on Nature Research policies, see our Editorial Policies and the Editorial Policy Checklist.

Statistics

For all statistical analyses, confirm that the following items are present in the figure legend, table legend, main text, or Methods section.

n/a	Confirmed	
☐	☒	The exact sample size (*n*) for each experimental group/condition, given as a discrete number and unit of measurement
☒	☐	A statement on whether measurements were taken from distinct samples or whether the same sample was measured repeatedly
☒	☐	The statistical test(s) used AND whether they are one- or two-sided<br>*Only common tests should be described solely by name; describe more complex techniques in the Methods section.*
☒	☐	A description of all covariates tested
☒	☐	A description of any assumptions or corrections, such as tests of normality and adjustment for multiple comparisons
☐	☒	A full description of the statistical parameters including central tendency (e.g. means) or other basic estimates (e.g. regression coefficient) AND variation (e.g. standard deviation) or associated estimates of uncertainty (e.g. confidence intervals)
☒	☐	For null hypothesis testing, the test statistic (e.g. *F*, *t*, *r*) with confidence intervals, effect sizes, degrees of freedom and *P* value noted<br>*Give P values as exact values whenever suitable.*
☒	☐	For Bayesian analysis, information on the choice of priors and Markov chain Monte Carlo settings
☒	☐	For hierarchical and complex designs, identification of the appropriate level for tests and full reporting of outcomes
☒	☐	Estimates of effect sizes (e.g. Cohen's *d*, Pearson's *r*), indicating how they were calculated

Our web collection on statistics for biologists contains articles on many of the points above.

Software and code

Policy information about availability of computer code

Data collection	AMBER 2018<br>Gaussian 09<br>Python packages: NumPy, SciPy
Data analysis	Python packages: NumPy, SciPy, SciKit-Learn<br>EXAT - EXcitonic Analysis Tool (https://zenodo.org/record/4282773)<br>quantarhei (https://github.com/slamavl/quantarhei.git)

For manuscripts utilizing custom algorithms or software that are central to the research but not yet described in published literature, software must be made available to editors and reviewers. We strongly encourage code deposition in a community repository (e.g. GitHub). See the Nature Research guidelines for submitting code & software for further information.

Data

Policy information about availability of data

All manuscripts must include a data availability statement. This statement should provide the following information, where applicable:

- Accession codes, unique identifiers, or web links for publicly available datasets
- A list of figures that have associated raw data
- A description of any restrictions on data availability

Representative structures of the four clusters are given in Supplementary Data 1.
The datasets generated during and/or analysed during the current study are available as Source Data File in Supplementary Data 2--16.
The raw data are provided for the figures: Figure 3, Figures 4--7, Supplementary Figures 2--4, Supplementary Figures 6--10, Supplementary Figure 12, Supplementary Figures 14--22 and Supplementary Table 4. The raw data for the Supplementary Tables 1-3 are included in the raw data for the Figure 5.

Field-specific reporting

Please select the one below that is the best fit for your research. If you are not sure, read the appropriate sections before making your selection.

☒ Life sciences ☐ Behavioural & social sciences ☐ Ecological, evolutionary & environmental sciences

For a reference copy of the document with all sections, see nature.com/documents/nr-reporting-summary-flat.pdf

Life sciences study design

All studies must disclose on these points even when the disclosure is negative.

Sample size	The number of replicas was determined by a reasonable compromise between sampling accuracy and computational feasibility.
Data exclusions	We excluded from spectral calculations all structures coming from MD6, due to the fortuitous interaction of N1-lut with the a603-a609 dimer, which we regard as unphysical and irrelevant for LHCSR1.
Replication	Six replicas were used plus two additional replicas after aMD equilibration.
Randomization	No randomization was performed as not relevant for the present study
Blinding	No blinding was performed as not relevant for the present study.

Reporting for specific materials, systems and methods

We require information from authors about some types of materials, experimental systems and methods used in many studies. Here, indicate whether each material, system or method listed is relevant to your study. If you are not sure if a list item applies to your research, read the appropriate section before selecting a response.

Materials & experimental systems

n/a	Involved in the study	
☒	☐	Antibodies
☒	☐	Eukaryotic cell lines
☒	☐	Palaeontology and archaeology
☒	☐	Animals and other organisms
☒	☐	Human research participants
☒	☐	Clinical data
☒	☐	Dual use research of concern

Methods

n/a	Involved in the study	
☒	☐	ChIP-seq
☒	☐	Flow cytometry
☒	☐	MRI-based neuroimaging
